# A review on molecular simulations for the rupture of cross-linked polymer networks

**DOI:** 10.1080/14686996.2025.2587391

**Published:** 2025-11-10

**Authors:** Yuichi Masubuchi, Takato Ishida, Yusuke Koide, Takashi Uneyama

**Affiliations:** Department of Materials Physics, Nagoya University, Nagoya, Japan

**Keywords:** Polymers, networks, simulations, rupture, fracture, toughness, strength, coarse-graining

## Abstract

Although simulation studies focused on polymer network rupture remain relatively limited compared to the broader field, recent advances have enabled increasingly nuanced investigations that bridge molecular structures and macroscopic failure behaviors. This review surveys the evolution of molecular simulation approaches for polymer network rupture, from early studies on related materials to state-of-the-art methods. Key challenges — including mismatched spatial and temporal scales with experiments, the validity of coarse-grained models, the choice of simulation protocols and boundary conditions, and the development of meaningful structural descriptors — are critically discussed. Special attention is paid to the assumptions underlying universality, limitations of current methodologies, and the ongoing need for theoretically sound and experimentally accessible network characterization. Continued progress in computational techniques, model development, and integration with experimental insights will be essential for a deeper, predictive understanding of polymer network rupture.

## IMPACT STATEMENT

This review provides the most comprehensive and up-to-date synthesis on molecular simulation approaches for the rupture of polymer networks, a subject for which no prior review literature exists in the database. It identifies persistent pitfalls and bottlenecks in the field to propose concrete future directions, addressing the pressing need for theoretical and methodological clarity as the community moves toward predictive modeling.

## GRAPHICAL ABSTRACT

**Figure:**
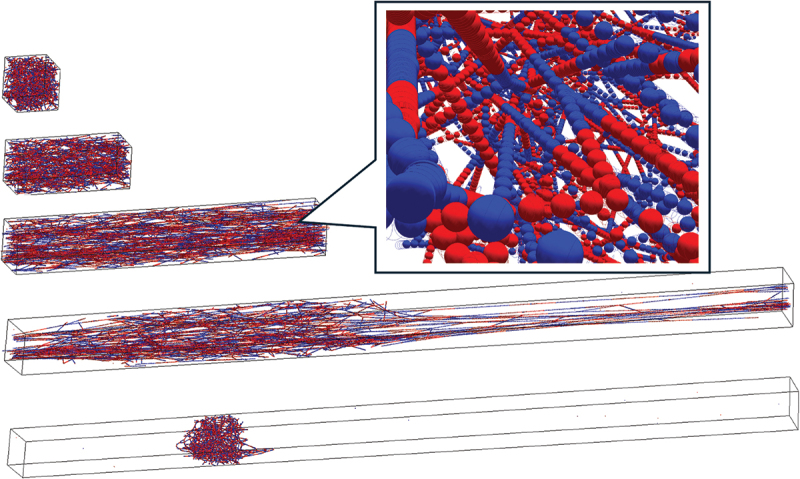

## 1. Introduction

**Figure 1.**
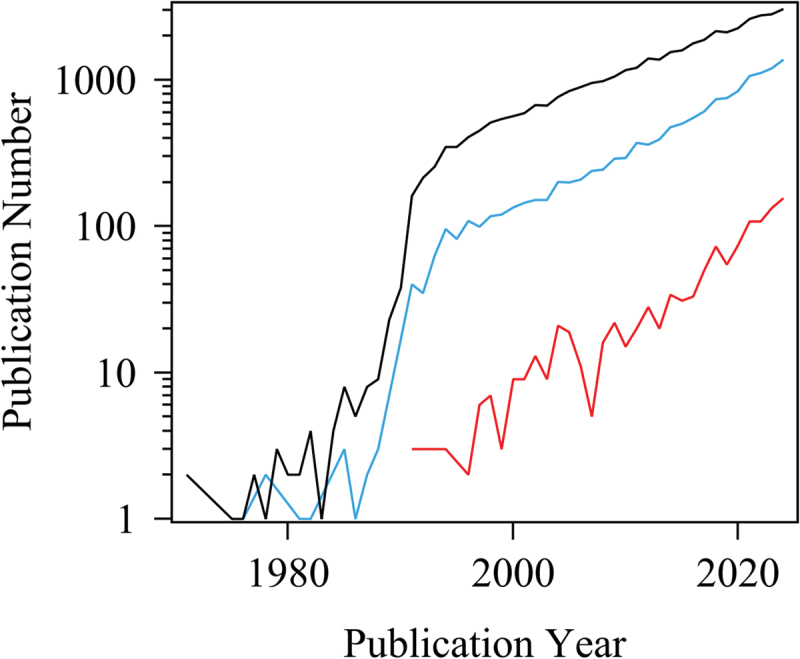
Annual number of publications on molecular simulations of polymers (black curve), polymer network simulations including rubbers, gels, and epoxies (blue curve), and simulations specifically addressing rupture and fracture of polymer networks (red curve), based on web of Science data as of June 2025. The data illustrate exponential growth in polymer simulation research, with rupture-focused studies emerging as a distinct but still developing area.

The interplay between the molecular structure of polymer networks and their mechanical properties, particularly fracture and rupture, remains a fundamental yet elusive issue in polymer science [1,2]. To elucidate this complex relationship, molecular simulations have become increasingly indispensable tools [3]. As illustrated in Figure 1, the annual number of publications on polymer simulations (black curve) has grown exponentially in recent decades, exceeding 3,000 per year. Among these, studies focused on polymer networks (blue curve) follow a similar upward trajectory, while those explicitly investigating polymer network rupture and fracture (red curve) – though fewer – are steadily increasing.

Despite these advances, simulating polymer network rupture at the molecular level remains challenging due to the inherent complexity of network architectures and the scale disparities between simulations and experiments. This review begins with an overview of the historical development of rupture simulations, contextualizing polymer network studies within broader efforts on related materials. It then addresses the key conceptual and technical challenges faced in simulating rupture phenomena, highlighting recent progress, limitations, and open questions that define the current research frontier.

## 2. Historical background

As explained later, molecular-level simulations of polymer network rupture have predominantly emerged since the early 2000s. These efforts build upon foundational studies from related fields that provide critical insights into fracture phenomena.

Atomistic simulations of crack tips in crystalline solids date back to the 1970s [4]. Thomson et al. [5] revealed how atomic discreteness creates energy barriers for the propagation of cracks in brittle solids. Sinclair and Lawn [6,7] combined continuum elasticity with atomistic relaxation to model crack-tip structures in diamond-type crystals. Such a direction was followed by other researchers, who focused on atomic-scale mechanisms, such as crack-tip plasticity [8–10].

Note that some studies in the 1980s employed similar lattice setups as atomistic simulations, but for different aims. Herrmann et al. [11–13] and Duxbury et al. [14,15] investigated the random fuse model, whereas Meakin [16,17] studied spring network models to simulate crack nucleation and propagation in brittle, disordered solids, thereby laying the groundwork for network-based approaches to fracture. This direction, considering the fracture of the modeled elastic body, has been widely explored [18,19].

Parallel to fracture studies, polymer dynamics simulations began evolving in the 1970s [20–22]. To accommodate the slow dynamics, coarse-grained bead-spring models were employed from these earliest studies. In the late 1980s, Brownian dynamics simulation for the bead-spring chain was established to reproduce polymer dynamics in melts [23–26]. Molecular dynamics simulations with united atom models were also developed [27]. Based on these models and methodologies, studies have been conducted on the yield behaviors of polymeric glasses under elongation [28,29]. Later, owing to the progress in computational technologies, full-atomistic models have also been employed for glassy polymers. For instance, Hutnik et al. [30] reported full-atomistic simulations of polycarbonate under plastic deformations, based on the methodology established by Theodorou and Suter [31]. Recent computational facilities have enabled further large-scale and long-duration simulations [32,33].

**Figure 2.**
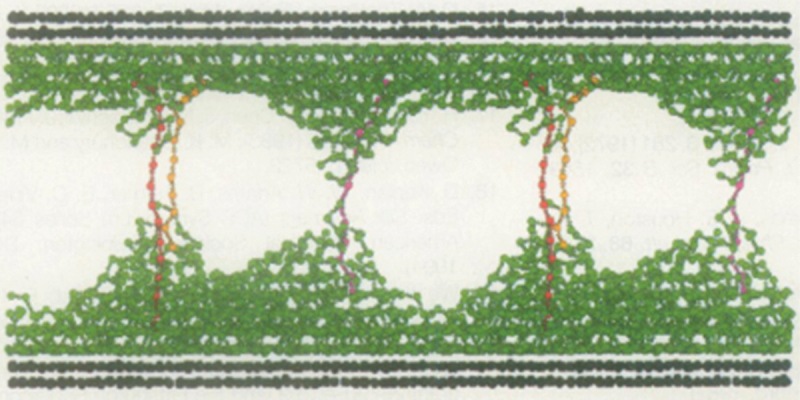
A snapshot of the melt rupture simulation between solid walls by Baijon and Robbins [34], with permission from the publisher. Copyright 1996, The American Association for the Advancement of Science.

In the 1990s, integrating the approaches mentioned above, Baijon and Robbins [34] introduced polymers into crack tip simulations to report apparent rupture of polymeric liquids. They placed melts of bead-spring chains between solid walls and observed the rupture of the melts as the distance between the walls increased, as shown in Figure 2. Robbins et al. [35,36] extended this approach to the fracture of polymer glasses. Similar studies on polymer nanocomposites [37] and end-grafted polymers attached to the wall surface [38,39] have also been conducted.

Building on this foundation, Stevens [40,41] pioneered rupture simulations of densely cross-linked epoxy-like networks in the 2000s. He introduced bond breakage and varied the interfacial bonding density between polymers and solid walls, and observed the transition between cohesive and interfacial failure. (This cohesive failure corresponds to the rupture of the stretched polymer network between walls.) Following his work, attempts have been made to extend the model towards complex and realistic systems. Tsige et al. [42,43] assessed the influence of cross-linker functionality. Subsequent modifications introduced ionic interactions [44] and bending rigidity [45,46]. The effects of entanglement have also been discussed [47]. As a simulation study in the early period, the work by Yarovsky and Evans [48] is also noteworthy because they constructed a full-atomistic model of epoxy attached to an alumina surface and calculated the adhesion energy, although cohesive failure is not discussed.

Eliminating the effect of the wall boundary, Rottler and Robins [49,50] investigated the fracture of bead-spring polymers in the glassy state by applying boundary conditions that stretched the system. Following their method, Panico et al. [51] investigate the effects of cross-link density on the fracture of glassy polymers. With similar simulation settings, Nouri et al. [52] conducted full-atomistic simulations for the fracture of epoxy networks. Full-atomistic modeling was also attempted for polybutadiene rubber [53] and polyurethane [54]. Moller et al. [55] investigated epoxy employing a united atom model. For bead-spring models, the effects of bending rigidity [56], entanglements [57,58], chain stiffness [59], and loops [60] have been discussed. Large-scale bead-spring simulations have been reported for bimodal networks [61], polymer nanocomposites [62,63], double-network systems [64], and slide-ring networks [65]. To consider the widely dispersed relaxation modes, simulations for vitrimers have been attempted with further coarse-grained models [66,67].

Due to critical spatial and temporal scale challenges in molecular simulations, continuum approaches have been pursued concurrently. Early work by Tijssens et al. [68] modeled crazing in polymer glasses via a finite element method. Later, Miehe et al. [69,70] applied the phase field modeling technique to rubbery polymers, and this approach has been further explored [71–73]. Since this review focuses on molecular simulations, further details on continuum approaches are left to other literature [74–76].

**Figure 3.**
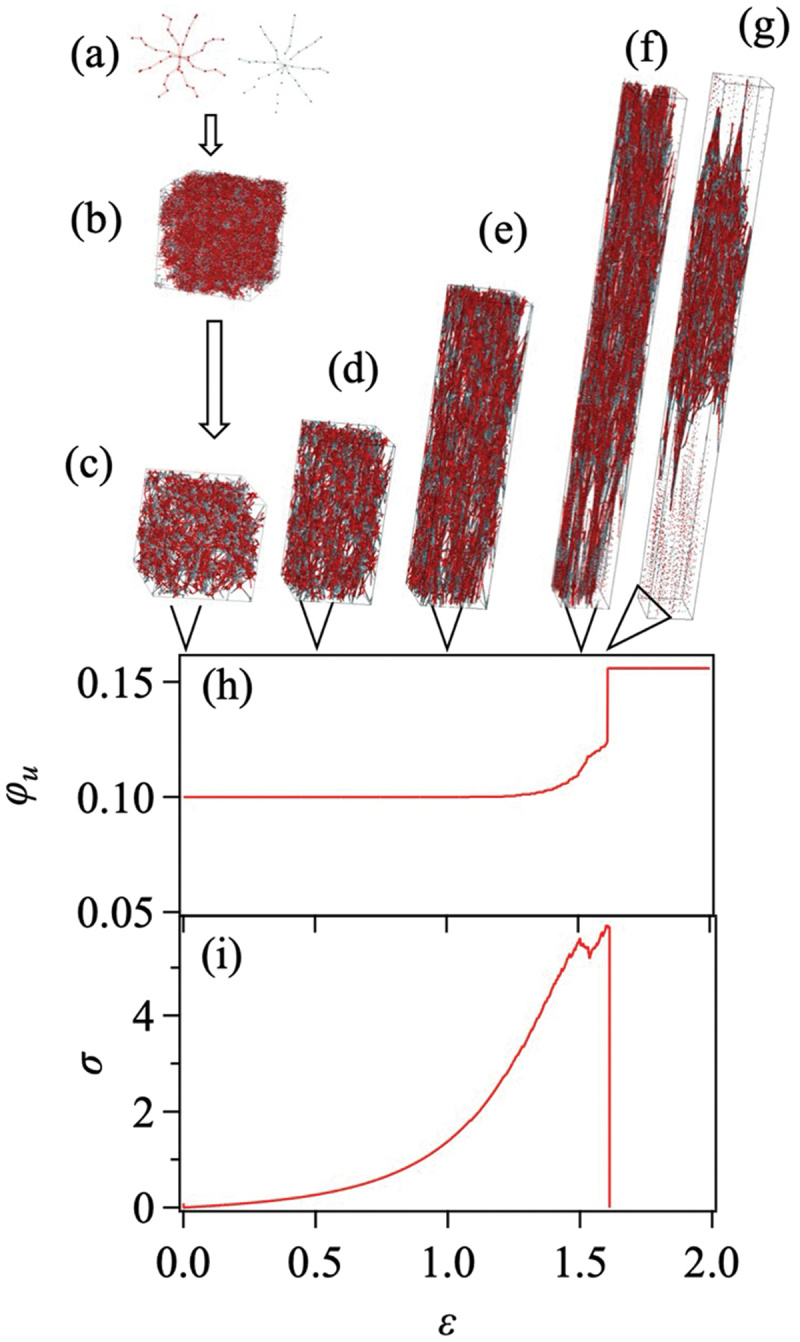
Typical snapshots in rupture simulations for phantom chain networks; prepolymers (a), the gelated network (b), the energy-minimized structure (c), the stretched states (d)–(f), the broken network (g), development of unconnected strand fraction $\varphi_{u}$ (h) and stress σ (i) during the stretch plotted against true strain ε. The prepolymer functionality f=8 and conversion $\varphi_{c}=0.9$ taken from Masubuchi et al. [88], with permission from the publisher, copyright 2023, American Chemical Society.

Complementary to these continuum and atomistic approaches are mesoscopic models incorporating explicit polymer connectivity while simplifying other aspects. Arora et al. [77–79] introduced such a model to discuss the effects of topological defects, including loops and dangling ends, and spatial inhomogeneity of network node density. Masubuchi et al. [80–88] investigated similar phantom chain networks to discuss the effects of strand length, its bimodality, node functionality, conversion, prepolymer concentration, and other factors. A typical example is shown in Figure 3.

## 3. System size and stretching conditions

A critical aspect of molecular simulations of polymer network rupture is the choice of system size and simulation duration, which must be sufficiently large and long to capture the relevant phenomena. Due to computational constraints, the dimensions of simulations and strain rates often differ substantially from those of experimental conditions. Although these difficulties are well-known and familiar in molecular simulations, they become especially critical in rupture simulations because the dominant temporal and spatial scales in rupture rapidly grow with time.

For example, Stevens [40,41] estimated the plastic zone size near a crack tip in epoxy to be approximately 10 μm, while his simulations with 170,000 beads represent a region smaller than 100 nm. More recent large-scale simulations with over 1.6 million beads [61] suggest that, at least for specific rupture characteristics, size effects may be limited; however, such generalizations depend heavily on the particular problem at hand. Notably, the simulation box size imposes artificial cutoffs on the probability distribution of fracture characteristics [89–92], a factor that is seldom discussed.

Temporal scaling presents an even greater challenge. In Stevens’ work [40,41], the stretch speed used for most cases was 10^−3^ in Lennard-Jones (LJ) units, which is comparable to the Rouse relaxation rate of a linear chain with 30 beads [26,93], but significantly faster than the relaxation rate of his entire network containing 170,000 beads. The study by Sliozberg et al. [57] employed a stretching speed of 10^−5^ in LJ units for their system with 500,000 beads. Yet, they stated that this stretch is much faster than in experiments, as explicitly indicated in the title, ‘high-strain rate deformation’, even for the systems including monomer beads as solvents. Even for recent simulations, the stretching speed remains to be higher than 10^−5^ in LJ units for most cases.

One may argue that the relaxation of the single strand is dominant in the relaxation of the network. This view is suitable for unbreakable rubbery networks [94–98]. In contrast, for network rupture and fracture, structural relaxation occurs after every single strand breakage. In a cascade of bond scission and macroscopic network failure, structural relaxation and mitigation propagate throughout the entire system, with a characteristic time that rapidly increases due to changes in network connectivity. For example, Brownian dynamics studies of phantom chain networks demonstrate that when strain rates exceed the reciprocal relaxation time of disconnected network domains, residual stresses persist even after macroscopic failure [84,85,87].

Note that most of the rupture simulations were made under constant stretch speed; the walls or the boundary of the simulation box are moved with a constant speed. This condition is consistent with most rupture experiments for polymeric solids and is fair when the effects of strain rate are negligible. In contrast, if the rupture behavior depends on the stretch speed reflecting the breakage of the network, deformation conditions under a constant Hencky strain rate would be appropriate for discussing the competition between relaxation and deformation, analogous to the extensional rheology of polymeric liquids. A few simulation studies explicitly state that they elongated the system with constant Hencky strain rates [60,84,85].

To alleviate the influence of strain rate, some studies employ quasi-static or energy minimization approaches that disregard dynamic effects, focusing instead on mechanical equilibrium and force balances [99–101]. Masubuchi et al. [80–83,86–88] employed this approach to observe network rupture, eliminating the effects of strain rate, as illustrated in Figure 3. The drawback is the lack of relaxation and energy dissipation [60].

Another often unaddressed but essential factor is the choice of elongational boundary conditions [102]. In simulations with solid walls [40–47], simulation box sizes in the lateral directions are unchanged, and the volume increases as the system is stretched. As mentioned by Baijon and Robbins [34], these studies aim to reproduce what happens at the crack tip in tearing tests, where the system size increases as deformation is applied. Some simulations without solid walls also employ this condition [52,64,65]. The other approach is to determine the system size based on pressure using NPT ensemble techniques [51,53–56,60]. The remaining simulations assume incompressibility, and the simulation box sizes in the lateral directions decrease as the elongation increases [58,61,78,85,87]. These simulations aim to replicate the behavior of bulk materials under tensile testing conditions. Since tearing and tensile tests experimentally probe different failure mechanisms, careful consideration of boundary conditions is crucial for meaningful comparisons between simulations and experiments.

Lastly, the definition of stress employed in simulations affects the interpretation of stress – strain relations [102]. Experimental fracture testing commonly reports nominal (engineering) stress for convenience, whereas molecular simulations calculate true stress from microscopic virial expressions [103,104]. When lateral dimensions are fixed, nominal and true stresses coincide; otherwise, appropriate conversions are necessary to maintain correct conjugacy with nominal strain during data analysis [105–108].

## 4. Coarse-graining

As mentioned above, the coarse-grained bead-spring model and its derivatives have been utilized due to their efficiency in reducing computational costs. However, constructing and validating coarse-grained models is not a trivial task [109–113]. The widely adopted bead-spring model by Kremer and Grest [24] is justified by its ability to reproduce key features of entangled polymer dynamics, which exhibit universality across different chemistries as demonstrated by extensive experimental evidence [114,115]. This universality has enabled further coarse-grained descriptions, such as tube models, to effectively capture the dynamics of polymers [116–118].

Simulations of polymer networks often build on these insights, assuming that chemistry-dependent effects can be subsumed into a limited set of model parameters associated with beads and springs. However, the universality of rupture phenomena across diverse chemistries remains unestablished. Thus, the widespread rationalization of coarse-grained models for rupture remains pending. Fine-grained atomistic simulations [52,53,55] provide complementary insights, although they face even steeper challenges in bridging spatial and temporal scales.

A frequently underappreciated issue concerns the equation of motion under deformation within coarse-grained modeling. Projection operator techniques [119,120] demonstrate that the eliminated degrees of freedom in coarse-graining act as effective drag and random forces, leading to Langevin [104,121] or dissipative particle dynamics (DPD) [122,123] equations of motion. However, rigorous coarse-graining theory for nonequilibrium, deforming systems remains lacking. In addition, while several nonequilibrium molecular dynamics methods exist [124], no thermostat is yet theoretically proven to dissipate deformation-injected energy under strongly nonequilibrium conditions correctly.

Consequently, equations of motion used in rupture simulations vary. In particular, modeling the background flow in Langevin dynamics is inconsistent: some studies neglect it, assuming a quiescent flow, while others account for it [84,85]. Given that deformation rates in typical rupture simulations exceed reciprocal relaxation times, neglecting background flow may introduce artifacts, such as the suppression of inhomogeneous void formation. Complex flow patterns inevitably develop near voids and interfaces, further complicating the modeling. Modified DPD schemes [125,126] have been proposed to address these issues, with promising results demonstrated in liquid rupture simulations [127].

## 5. Network structure

A critical aspect of polymer network rupture simulations is the design and characterization of the network itself. Widely adopted approaches construct networks by mimicking experimental methods, such as the polymerization of small molecules [40–46,52,54–56,58,64], cross-linking of linear prepolymers in melts or solutions [47,51,53,57], end-linking of star polymer precursors [47,51,53,57], and cross-linking linear prepolymers with multifunctional linkers [59,78,82]. These methodologies largely build upon earlier foundational studies [128–130].

However, the network structures generated in simulations may differ from actual experimental materials due to inherent challenges in replicating reaction kinetics and gelation timescales. Kinetic arrest during gelation [131,132] commonly alters the network topology, and simulating these dynamic processes accurately is challenging due to the mismatched time domains between simulations and experiments. Consequently, rigorous evaluation of the simulated network structure is necessary. This task is inherently circular: dominant structural descriptors for rupture should guide network design, yet identifying such descriptors often requires analyzing the network post-simulation. Moreover, experimental characterization of network topology remains an evolving field [133].

Assuming the created networks approximate experimental systems, efforts have focused on identifying the key structural parameters that govern rupture. Early work by Stevens [40,41] emphasized the shortest path in the stretching direction as an important descriptor, a concept recently refined by Yu and Jackson [134]. Rooted in the Lake-Thomas theory [135], classical parameters such as network node density and node functionality continue to play central roles in network analysis. Extending this approach, Barney et al. [60] investigated the influence of loop fractions on fracture energy, connecting simulations to experiments via the theoretical model [136]. Recently, cycle rank – a topological quantity representing the density of independent loops – has been proposed as an effective descriptor that unifies influences of node functionality and conversion [88]. This metric is amenable to estimation using mean-field theories [137,138], enabling experimental applicability [139]. Yang and Qu [56] discussed the formation of cavities in epoxy in relation to rupture. Zhang and Riggleman [140] investigated network failure using geodesic edge betweenness centrality, building on previous studies in 2D systems [141,142].

**Figure 4.**
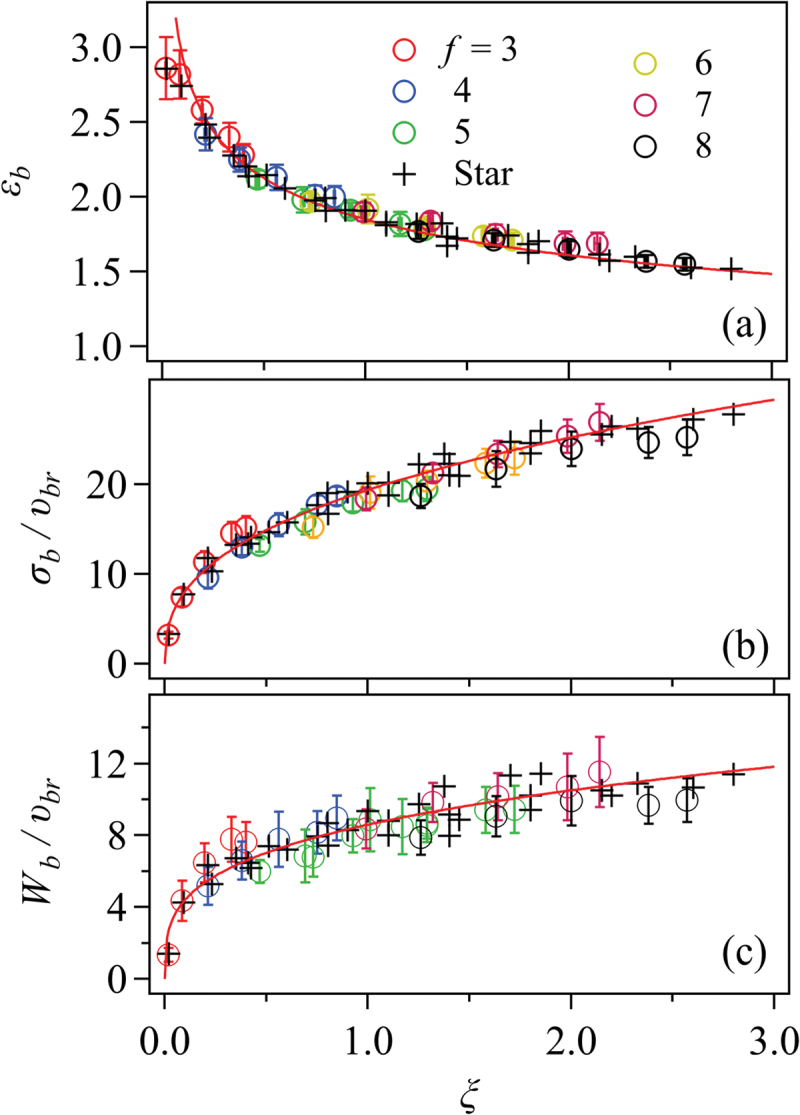
Strain at break $\varepsilon_{b}$ (a), stress at break $\sigma_{b}$ (b), and work for rupture $W_{b}$ (c) plotted against cycle rank ξ for phantom chain networks with various node functionality f and conversions between 0.6 and 0.95. The strand segment number is 10, and the strand density is 8. The circles show the results from the systems created from linear prepolymers and multi-functional linkers, whereas the cross shows those for star prepolymers.

Most of these studies implicitly assume a degree of universality across chemistries by employing coarse-grained models. For instance, the rupture characteristics for phantom chain networks with varying node functionalities and conversions, but identical strand lengths, collapse onto master curves when plotted against cycle rank density [82,88] (Figure 4). Although promising, this universality remains unverified for chemically diverse systems.

Besides, there is an open problem regarding universality across structurally distinct network classes. For example, networks based on regular lattice topologies or graph theory may exhibit rupture behavior that differs fundamentally from that of random or statistically generated networks, highlighting the need for further investigation [143–145].

## 6. Summary

Molecular simulations have become indispensable for unraveling the rupture behavior of polymer networks, offering microscopic insight into phenomena that are challenging to access experimentally. Nevertheless, progress in this field continues to be hampered by several fundamental difficulties. One persistent challenge is the significant mismatch in spatial and temporal scales between simulations and experiments. While advances in computational power and coarse-grained modeling have enabled larger and longer simulations, key assumptions – such as the universality of rupture behavior across different chemistries – have yet to be systematically validated. This limitation is particularly significant for rupture phenomena, as local chemistry and network topology can both profoundly influence fracture response. Equally important is the careful selection and transparent reporting of simulation parameters, including system size, deformation protocol, boundary conditions, and the definitions of stress and strain. These factors critically impact the interpretation of simulation results and their applicability to real-world applications. Similarly, the impact of coarse-graining strategies and the choice of equations of motion or thermostats in nonequilibrium conditions must be scrutinized for their effect on the fidelity of rupture simulations. Due to the difficulties outlined above, there is no established simulation methodology; therefore, significant theoretical and technical developments remain needed. Besides the technical challenges, another major unresolved issue is the development and validation of robust, theoretically sound, and experimentally accessible descriptors of network structure. While each simulation study reviewed in this article reported valuable results for each system, the lack of universality makes it challenging to have a unified discussion across different systems. In addition, direct and meaningful comparison between simulations and experimental systems also remains elusive. In sum, while molecular simulations have shed light on the rupture of polymer networks, continued progress will require both methodological innovations and more precise, theory-driven characterization techniques to close the gap between model predictions and experimental observations.
